# Supplementary material for: Potent Protective Immune Responses to Senecavirus Induced by Virus-Like Particle Vaccine in Pigs
Source: Vaccines (Basel). 2020 Sep 15;8(3):532. doi: 10.3390/vaccines8030532 (PMC7565160; doi:10.3390/vaccines8030532)
Supplement: Supplementary file 1 [file vaccines-08-00532-s001.zip › vaccines-874957-proof/Supplementary information.docx]

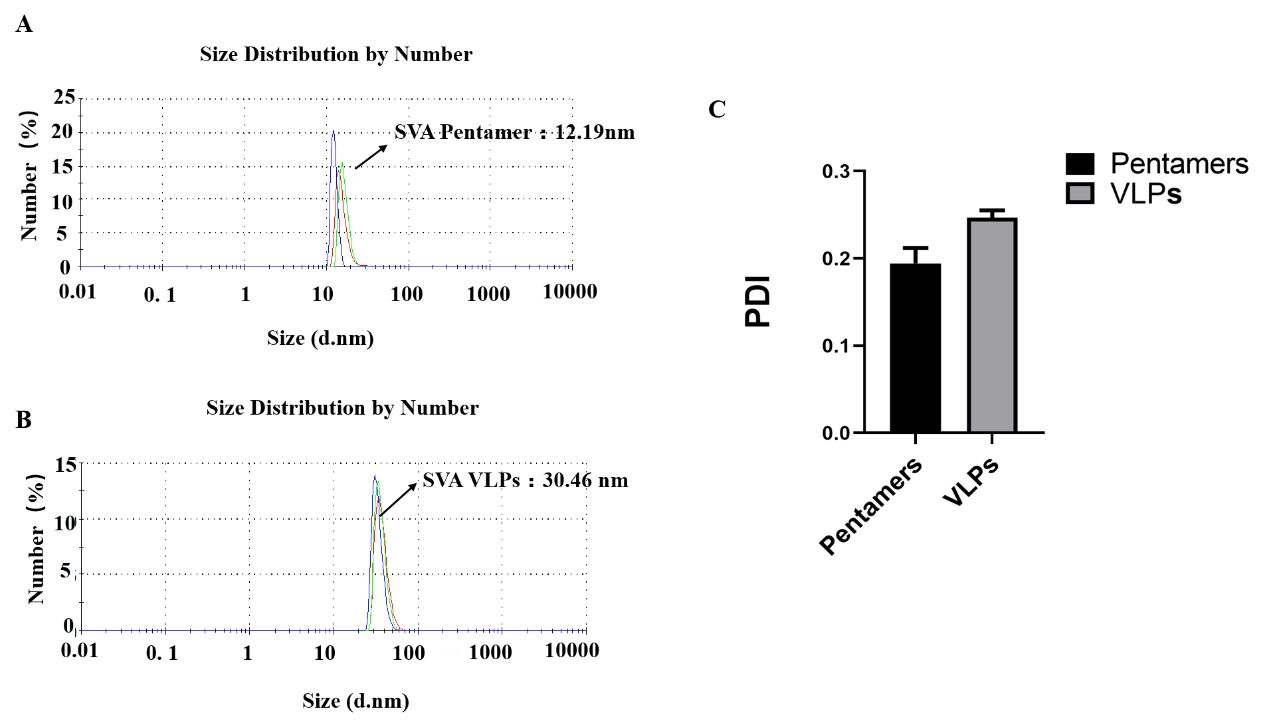


**Supplementary Figure:.Z**etasizer Nano measurements of Pentamer and SVA VLPs.

**(A and B): Particle size were shown in the peak sample of 11 tubes (Pentamer) and peak sample of 16 tubes (VLPs).** (C) The polydispersity index (PDI) of the measurements.
