# Supplementary figures and images for: Potent Protective Immune Responses to Senecavirus Induced by Virus-Like Particle Vaccine in Pigs
Source: Vaccines (Basel). 2020 Sep 15;8(3):532. doi: 10.3390/vaccines8030532 (PMC7565160; doi:10.3390/vaccines8030532)

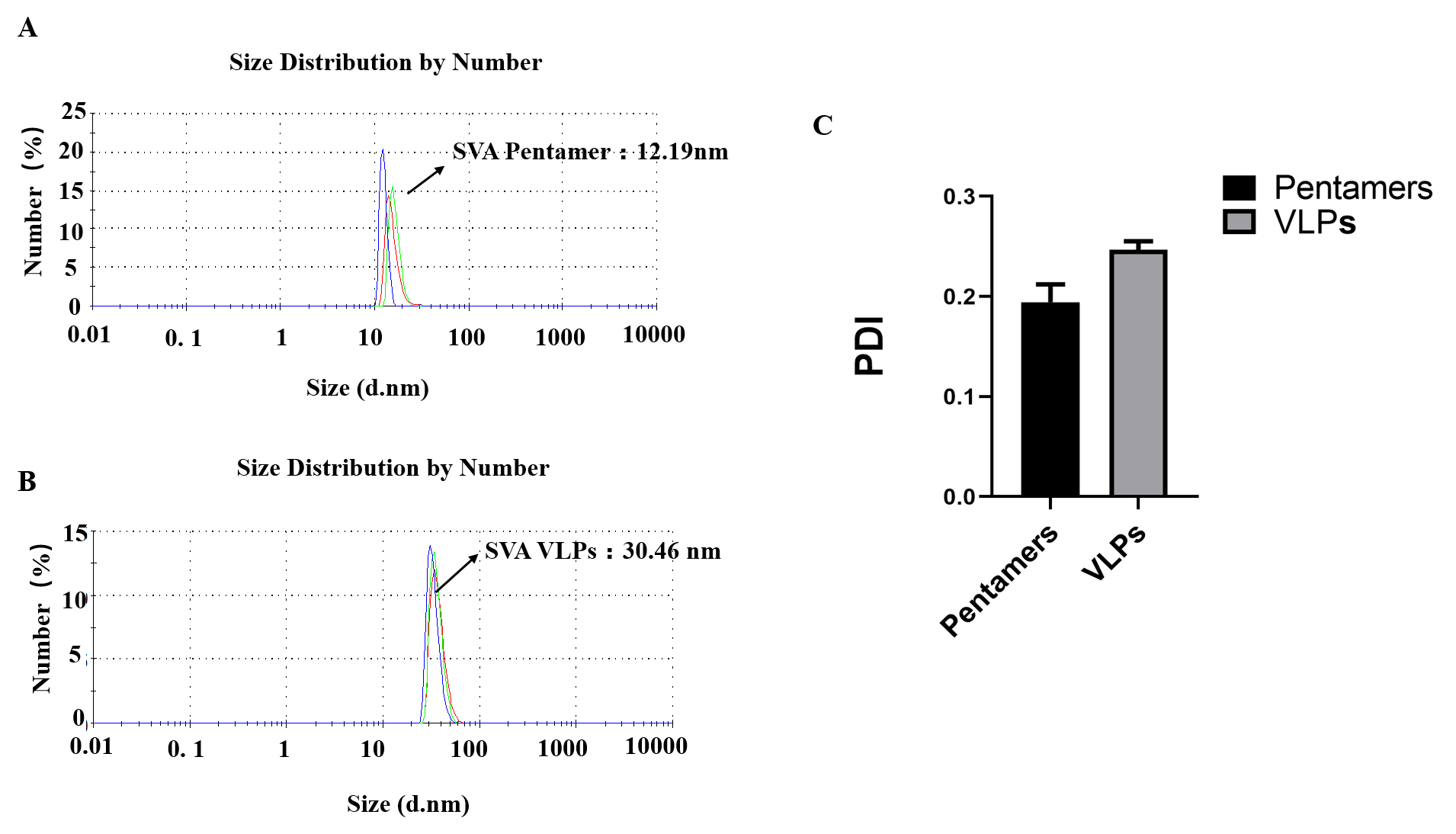

Supplement: Supplementary file 1 [file vaccines-08-00532-s001.zip › vaccines-874957-proof/Supplementary information.tif]
